# Biomarker Genes for Detecting Estrogenic Activity of Endocrine Disruptors via Estrogen Receptors

**DOI:** 10.3390/ijerph9030698

**Published:** 2012-02-24

**Authors:** Eui-Man Jung, Beum-Soo An, Hyun Yang, Kyung-Chul Choi, Eui-Bae Jeung

**Affiliations:** Laboratory of Veterinary Biochemistry and Molecular Biology, College of Veterinary Medicine, Chungbuk National University, Cheongju, Chungbuk 361-763, Korea; Email: jemman@hanmail.net (E.M.J.); vetterang@hotmail.com (B.S.A.); anpoong@naver.com (H.Y.); kchoi@chungbuk.ac.kr (K.C.C.)

**Keywords:** endocrine disruptors, estrogen activity, biomarker, calbindin-D_9k_

## Abstract

Endocrine disruptors (EDs) are compounds used in various industrial products, drugs, and cosmetics. They can be found in the environment and disturb the endocrine and reproductive systems, resulting in adverse effects to humans and wildlife such as birth defects and developmental disorders. Since several EDs have a structure similar to that of endogenous steroid hormones such as estrogens, they intend to have an affinity for steroid hormone receptors and alter hormone-mediated metabolism by binding to these receptors. EDs are therefore a global concern and assays should be developed to efficiently determine whether these compounds are detrimental to biological systems. Diverse experimental methods may help determine the endocrine disrupting potential of EDs and evaluate the adverse effects of a single and/or combination of these reagents. Currently, biomarkers have been employed to objectively measure EDs potency and understand the underlying mechanisms. Further studies are required to develop ideal screening methods and biomarkers to determine EDs potency at environmentally relevant concentrations. In this review, we describe the biomarkers for estrogenicity of EDs identified both *in vitro* and *in vivo*, and introduce a biomarker, cabindin-D_9k_ (CaBP-9k), that may be used to assess estrogenic activity of EDs.

## 1. Introduction

Endocrine disruptors (EDs) are environmental chemicals that disrupt the endocrine system of wildlife and humans. Studies performed in the 1930s showed that a number of synthetic chemicals have estrogenic properties, and the feminizing effect of the pesticide dichlorodiphenyltrichloroethane (DDT) was demonstrated in roosters in the 1950s [1,2]. Hormonally active chemicals, (*i.e.*, diethylstilbestrol or DES) were widely used to prevent abortions until 1971; however, vaginal clear cell carcinogenesis was observed in young women due to use of synthetic estrogen during pregnancy [3]. Furthermore, female offspring from women who were exposed to DES had an increased risk of reproductive and immunologic abnormalities, and male offspring were at risk for genital anomalies and abnormal spermatogenesis [4,5]. It was also reported that EDs potentially cause fetal pathophysiology, abnormal maturation of the brain, learning disabilities, disorders associated with attention, motivation, emotion and cognitive development. In addition, reduction of sperm count and prostate enlargement in males; ovarian and uterine dysfunctions in females have been reported [6,7]. These findings indicate that hormonal disruption caused by EDs in animals and humans results in serious adverse effects.

EDs are widely found in drugs, pesticides, and compounds used for manufacturing plastics (see Table 1). Most EDs have a structure similar to that of endogenous steroid hormones and primarily act through nuclear hormone receptors including estrogen receptors (ERs), androgen receptors (ARs), and thyroid receptors (TRs) [8]. Having a structure similar to that of hormones permits EDs to exert abnormal effects that disrupt endocrine systems through diverse pathways including binding to hormone receptors, mimicking hormone action, and blocking the action of hormones [9,10,11]. Estrogen (E2) is hormone essential for the development of reproductive organs, bone, liver, and the cardiovascular system, and plays an important role in many physiological processes [12]. Diverse types of EDs have been shown to have estrogenic activity because of high binding affinity for ERs. ERs are steroid hormone nuclear receptors and act as ligand-activated transcription factors. ERs have two isoforms, ER-α and ER-β, which can bind a wide variety of EDs and activate the transcription of estrogen-responsive genes [13,14]. However, there are no standard methods to determine whether certain EDs have an estrogenic activity or not. In this review, we discuss diverse methods to evaluate EDs and discuss the use of CaBP-9k as a biomarker for identifying estrogenic EDs.

**Table 1:** List of estrogenic EDs.

Chemical Class	EDs	References
Industrial byproducts	Polychlorinated biphenyls (PCBs)	
	Polybrominated biphenyls (PBBs)	
	Dioxins	[15,16]
Plastics	Bisphenol A (BPA)	[17,18]
Plasticizers	Phthalates	[19,20]
Pesticides	Methoxychlor	
	Chlorpyrifos	
	Dichlorodiphenyltrichloroethane	[21,22]
Fungicides	Vinclozolin	[23,24]
Pharmaceutical agents	DES	[3,5]
Biodegradation products	Octyl-phenol (OP)	
	Nonylphenol (NP)	[25,26]
Phytoestrogens	Genistein	
	Coumestrol	[24,27,28]

## 2. Biomarkers for Measuring the Estrogenic Effect of EDs

### 2.1. Vitellogenin

Vitellogenin (VTG) is an egg yolk precursor protein and is normally produced by liver cells of female fish in response to E2 secreted by the pituitary gland [29]. It is released into the blood where it circulates until it reached the ovaries and promotes oocyte development. Male fish also carry the *VTG* gene, although VTG protein is normally not expressed because the circulation levels of E2 are extremely low in male blood plasma [30]. However, males still have the capability to express VTG, and male fish are known to produce the protein under the influence of estrogenic EDs [30,31,32]. E2, NP, and OP all induce the expression of VTG in male fish in a dose-dependent manner [33,34], suggesting that the *VTG* gene in male fish can be used as a biomarker for evaluating the effects of EDs [35,36,37]. 

### 2.2. Complement C3 and Ornithine Decarboxylase

The uterus is a general target organ for estrogen-mediated metabolism. A uterotrophic bioassay is widely used to measure increased uterine wet weight after EDs treatment [38]. However, this assay does not evaluate effects other than those associated with the estrogenic activity of EDs which could lead increased uterine weight via unknown pathways. In the past few years, several genes regulated in the uterus have been identified and used as marker genes to assess the estrogenicity of EDs. For example, genes for the gap junction connexin, such as connexin 26 and connexin 43, the plasma glycoprotein clusterin, and complement C3 were shown to be highly regulated by E2 in rat endometrium [39,40,41]. 

Complement C3, simply called C3, is a protein involved in the immune system which plays a central role in activating complement pathways and promotes innate immunity [42]. In adult female mice, C3 is exclusively expressed in the uterus. E2 administration to immature or ovariectomized mice significantly increases *C3* mRNA levels as well as immunoreactivity in the endometrium, indicating that the synthesis of this protein is regulated by E2 in mouse endometrium [43]. Phyto- and xenogestogens have been found to induce C3 expression in endometrium, and the sensitive parameter of C3 is highly suited to investigate the biological potential of natural and synthetic estrogens [39]. 

The enzyme ornithine decarboxylase catalyzes the decarboxylation of ornithine (a product of the urea cycle) to form putrescine, which is the committed step in polyamine synthesis [44]. The rapid growth and differentiation of uterus are concomitant with increased expression of the ornithine decarboxylase gene. Recent studies have shown that expression of ornithine decarboxylase gene in the uterus is augmented by EDs [45,46,47,48]. These estrogen-sensitive genes have therefore been used as markers for evaluating the estrogenic potential of EDs in the uterus

### 2.3. pS2 and Mucin 1

pS2 is a low molecular weight protein containing 60 amino acid. E2 and estrogenic compounds stimulate the expression of pS2 which was first observed in the MCF-7 breast cancer cell line into which the *pS2* gene has been cloned [49]. *pS2* mRNA production can be rapidly induced by E2 in certain breast cancers, but not in normal breast tissue nor in any other human cell lines. Therefore, *pS2* mRNA expression in MCF-7 cells is an ideal model for studying the effects of estrogenic compounds [49,50,51]. 

Cell surface mucins are a family of highly glycosylated glycoproteins found in the apical cell membranes of epithelial cells from the mammary gland, salivary gland, respiratory tract, digestive tract, uterus, and testis [52,53]. Mucin1 (MUC1), a mucin and well-known marker of breast cancer, is an extended rod-like molecule which protrudes above the cell surface of epithelial cells [54]. The MUC1 promoter region has half of an estrogen response element (ERE) and is regulated by E2. Therefore, MUC1 is known to be a direct E2 target gene due to specific ER binding in MCF-7 cells [55]. EDs, including NP, have been reported to induce the expression of pS2 and MUC1 in MCF-7 breast cancer cells [54]. 

### 2.4. Progesterone Receptor

The progesterone receptor (PR) is an intracellular steroid receptor that specifically binds to progesterone and is involved in a wide variety of physiological functions including the control of embryonic development, cell differentiation, and homeostasis [56,57,58]. The *PR* gene is a known target of E2 in certain cell lines including MCF-7 and GH3 cells that express ERs [59,60]. Recent studies have observed EDs-induced expression of PRs in GH3 rat pituitary cells. Several parabens and E2 significantly increased PR expression in these cells [60], demonstrating that PR levels may be augmented by EDs through an ER-mediated pathway in GH3 cells [61,62]. By measuring the expression levels of VTG, Complement C3, ornithine decarboxylase, pS2, MUC1 and PR genes, EDs estrogenicity can be efficiently evaluated in a cost- and time-effective manner. 

## 3. *CaBP-9k* Gene Expression as a Biomarker for Assessing the Effects of EDs

### 3.1. Introduction of the CaBP-9k Gene

Calbindin-D_9k_ (CaBP-9k) is a 9-kDa cytosolic protein and a member of the S100 family of calcium-binding proteins that includes calmodulin, paravalbumin, troponin C, and calbindin-D_28k_. These calcium-binding proteins were recently classified into different sub-families as they differ in the number of calcium-binding EF-hand sites [63,64]. CaBP-9k has two calcium-binding domains that have high affinities for calcium. The *CaBP-9k* gene is localized on the X chromosome and consists of three exons interrupted by two introns [65,66]. The CaBP-9k protein is composed of 79 amino acids. The *C*-terminal site (amino acids 54–65) is a normal EF-hand similar to that found in other proteins of the S100 family while the *N*-terminal site (amino acids 14–27) has a unique structure containing different amino acids. However, *N*-terminal site have not disrupted the calcium binding ability of CaBP-9k [67]. 

### 3.2. Regulation of CaBP-9k Gene Expression

CaBP-9k is an intracellular factor primarily known as a vitamin D-dependent calcium-binding protein found in the cytoplasm of intestinal cells [68,69]. CaBP-9k expression has been reported in intestine, kidneys, bone, lung, placenta, pituitary gland, and uterus [70]. Duodenal and renal CaBP-9k are involved in calcium absorption and re-absorption; its expression is regulated by 1,25-dihydroxyvitamin D3 [71,72]. Duodenal CaBP-9k is mainly expressed in enterocytes of the duodenum in rodents and is involved in intestinal calcium absorption [73]. Additionally, the level of *CaBP-9k* mRNA is thought to be a marker of small intestine differentiation [74,75]. Renal CaBP-9k plays a role in calcium re-absorption in the kidney, which is important for maintaining calcium homeostasis in the body. Ionized and calcium enters the glomerular filtrate by convection and is re-absorbed by the renal tubules [72]. Renal CaBP-9k is expressed at the site of calcium re-absorption and localized in the distal convoluted tubules in rodents [76,77]. 

The regulation of CaBP-9k expression in other tissues other than duodenum and kidney is known to be accomplished through different mechanisms. For instance, uterine CaBP-9k is not under the control of 1,25-dihydroxyvitamin D3 despite the presence of 1,25-dihydroxyvitamin D3 receptors [78]. Uterine CaBP-9k is expressed mainly in the endometrial stroma and myometrium in non-pregnant rodents and regulated by sex steroid hormones [79,80]. An estrogen response element (ERE) in the CaBP-9k promoter mediates transcriptional regulation of CaBP-9k in the presence of E2 in rat uterus [81]. On the other hand, a progesterone response element (PRE) in the CaBP-9k promoter is responsible for responsiveness to progesterone (P4) in mouse uterus [82]. Interestingly, uterine CaBP-9k is up-regulated by E2 and down-regulated by P4 during the estrous cycle in rat uterus [83,84], while it has been shown to be decreased by E2 in mouse uterus [85,86,87].

### 3.3. *In Vivo* Evaluation of Estrogenic Activity of EDs Using the CaBP-9k Gene

Recently, *CaBP-9k* was suggested to be a novel biomarker for evaluating EDs activity [88,89]. *CaBP-9k* expression levels increased by exposure to EDs are considered to be effective tools for screening estrogenic compounds in *in vivo* models. Recent studies demonstrated that estrogenic compounds significantly increase the expression of uterine CaBP-9k in rats [78]. An *et al*. showed that E2, OP, NP, and BPA increased uterine *CaBP-9k* gene expression in rats. Furthermore, these estrogenic compounds also significantly increase uterine weight in rats [84,89]. Estrogenic compounds, which form a class of EDs that includes diethylstilbestrol [90], tetrabromodiphenyl ether [91], phthalates [92] genistein [93], and parabens [94], strongly induce uterine *CaBP-9k* mRNA and protein expression in the uterus of rats. Moreover, the ER antagonist ICI 182,780 prevents increased expression of uterine *CaBP-9k* when co-administered with estrogenic compounds. It has been confirmed that ER-mediated signaling is involved in the induction of *CaBP-9k* gene expression by estrogenic compounds [95]. These findings suggest that the *CaBP-9k* gene is a useful biomarker gene for assessing the estrogenic potential of EDs.

The ability of *CaBP-9k* for evaluating EDs in an *in vivo* system is summarized in Table 2. Triclosan, tetrabromodiphenyl ether 47 (BDE47), and genistein at concentrations less than 50 mg/kg have been found to have regulatory effects on uterine CaBP-9k expression [91,93]. Triclosan at a dose of 37.5 mg/kg up-regulates uterine *CaBP-9k* expression in an immature rat model [96]. Genistein has similar potential with triclosan at dose of 40 mg/kg. Treatment with BDE47 (50 mg/kg) led a significant increase in uterine CaBP-9k as determined by RT-PCR. OP, NP, and BPA (600 mg/kg) induce uterine *CaBP-9k* expression at in the rat; however, lower concentrations (100 to 250 mg/kg) of these compounds had a more prominent effect in mice [84]. Methoxychlor (250 mg/kg) was reported to have a moderate effect on *CaBP-9k* expression in rat uterus [97]. Butyl benzyl phthalate (BBP), dicyclohexyl phthalate (DCHP), 2-ethylhexyl phthalate (DEHP), di-*n*-butyl phthalate (DBP), and diethyl phthalate (DEP) do not have any significant estrogenic effects on *CaBP-9k* gene expression in rat uterus [92]. Changes in CaBP-9k expression for assessing EDs activity are sufficient when compared to other biomarkers. For example, C3, a well-known biomarker for measuring EDs effects, shows a level of sensitivity similar to that of CaBP-9k at a concentration in the presence of OP and BPA (200 mg/kg) [40]. 

**Table 2:** Expression of uterine *CaBP-9k* in *in vivo* system by different EDs concentrations.

Endocrine disruptors	Doses (mg/kg)	Animal	Route	Method	Reference
Triclosan	37.5	rat	Oral	q-PCR/western	[96]
Genistein	40	rat	S.C injection	Western	[93]
Tetrabromodiphenyl ether 47	50	rat	S.C injection	RT-PCR	[91]
Octyl phenol	100/600	mouse/rat	S.C injection	Western	[84]
Methoxychlor	200	rat	Oral	Northern	[97]
Nonyl phenol	250/600	mouse/rat	S.C injection	Western	[84]
Bisphenol A	250/600	mouse/rat	S.C injection	Western	[84]

### 3.4. *In Vitro* Evaluation of Estrogenic Activity of EDs Using the *CaBP-9k* Gene

In 2004, Fujimoto *et al.* identified estrogen-responsive genes in the rat pituitary GH3 cell line [98]. GH3 cells were initially characterized as ones that can synthesize growth hormone and prolactin (PRL) in response to E2 stimulation [99]. It was suggested that the GH3 cell line is a good candidate for assessing the *in vitro* estrogenicity of EDs since it is E2-sensitive and expresses functional ERs [98]. Recent studies have reported that EDs induce CaBP-9k expression in this cell line. OP, NP, and BPA induce a significant increase in *CaBP-9k* expression in GH3 cells in addition to augmenting growth hormone and PRL production and gene expression [100]. Moreover, activation of extracellular signal-regulated kinases (ERKs), protein kinases B (Akt), or G proteins by OP, NP and BPA has been observed in these cells [99,100]. Significant induction in *CaBP-9k* and *PR* gene expression was also observed after treatment with various concentrations of parabens [61]. In this study, the effects of parabens on the regulation of *CaBP-9k* expression were blocked in the presence of ICI 182,780, indicating that CaBP-9k is induced by EDs via the ER pathway in GH3 cell line. 

Evaluation of the *CaBP-9k* gene as a biomarker for EDs activity in *in vitro* systems is summarized in Table 3. OP, isobutyl phenol, and ethyl- and isobutyl parabens produce the strongest induction of CaBP-9k expression at a concentration of 0.1 μM in GH3 cells, while NP and isopropyl- and butyl-parabens increase *CaBP-9k* gene expression at a concentration of 1 μM [100]. The concentration of BPA and methyl-and propyl-parabens required for inducing *CaBP-9k* gene expression is 10 μM [61]. The efficiency of *CaBP-9k* gene for EDs effect was sensitive than other genes, since pS2, ER and MUC1 were induced at 10 μM of NP in human breast MCF-7 cells, although the cell lines and other experimental conditions were different [54]. 

**Table 3:** Expression of *CaBP-9k* on *in vitro* system under different EDs concentration.

Endocrine disruptors	Minimum concentration (μM)	Method	Reference
Octyl phenol	0.1	q-PCR	[62]
Ethyl paraben	0.1	q-PCR	[101]
Isobutyl paraben	0.1	q-PCR	[101]
Nonyl phenol	1	RT-PCR	[100]
Isopropyl paraben	1	q-PCR	[101]
Butyl paraben	1	q-PCR	[101]
Bisphenol A	10	RT-PCR	[100]
Methyl paraben	10	q-PCR	[101]
Propyl paraben	10	q-PCR	[101]

The sensitivity of the *CaBP-9k* gene for assessing estrogenic activity of EDs compared to other biomarkers is difficult to define because effect of intermediate concentrations (*i.e.*, OP: 0.01–1 μM) of EDs have not been tested, particularly in *in vivo* models. However, the *CaBP-9k* gene has been evaluated as a biomarker for EDs activity by a number of *in vivo* and *in vitro* studies as described above. Taken together, data from these studies have demonstrated that the *CaBP-9k* gene is a sensitive and valuable biomarker for assessing the potential estrogenic activity of EDs.

## 4. Conclusions

EDs are natural or synthetic chemicals with structures similar to those of endogenous hormones and have been shown to disrupt endocrine, nervous, and reproduction systems in animals and humans. There are growing concerns about health problems associated with exposure to EDs found in the environment. Chemicals that disrupt the endocrine system have been shown to have a high binding affinity to steroid hormone receptors, suggesting that exposure to EDs can seriously impact metabolism, development, reproduction, and behavior in mammals, including humans. Therefore, identification and characterization of EDs is urgent for predicting their detrimental effects. Efficient and accurate assays are required to measure EDs potency and to determine what chemicals can possibility be defined as EDs. However, methods for detecting the adverse effects of EDs are still limited. There are currently no sufficient standard biomarker systems or precise methods for evaluating the effects of EDs. Further research to determine the physiological concentration of various EDs (administered alone or in combination) necessary for disturbing biological systems and exerting adverse effects on human health is required. In this review, we discussed the properties of the *CaBP-9k* gene and its potential use as a biomarker for EDs activity. However, further studies to examine the accumulated, synergistic, and addictive effect of EDs using this biomarker are needed. In addition, identification of novel biomarkers that can determine the biologically and physiologically relevant concentrations of EDs is necessary. 

## References

[1] Burlington H., Lindeman V.F. (1950). Effect of DDT on testes and secondary sex characters of white leghorn cockerels. Proc. Soc. Exp. Biol. Med..

[2] Poliakov A.I., Kriger M.S. (1961). Dichlorodiphenyltrichloroethane in the treatment of males with trichomonal urethritis. Vestn. Dermatol. Venerol..

[3] Schrager S., Potter B.E. (2004). Diethylstilbestrol exposure. Am. Fam. Physician.

[4] Giusti R.M., Iwamoto K., Hatch E.E. (1995). Diethylstilbestrol revisited: a review of the long-term health effects. Ann. Intern. Med..

[5] Yamamoto M., Shirai M., Tamura A., Kobayashi T., Kohara S., Murakami M., Arishima K. (2005). Effects of maternal exposure to a low dose of diethylstilbestrol on sexual dimorphic nucleus volume and male reproductive system in rat offspring. J. Toxicol. Sci..

[6] Bonde J.P., Storgaard L. (2002). How work-place conditions, environmental toxicants and lifestyle affect male reproductive function. Int. J. Androl..

[7] Calafat A.M., Kuklenyik Z., Reidy J.A., Caudill S.P., Ekong J., Needham L.L. (2005). Urinary concentrations of bisphenol a and 4-nonylphenol in a human reference population. Environ. Health Perspect..

[8] Korach K.S., Chae K., Gibson M., Curtis S. (1991). Estrogen receptor stereochemistry: ligand binding and hormonal responsiveness. Steroids.

[9] Kuiper G.G., Lemmen J.G., Carlsson B., Corton J.C., Safe S.H., van der Saag P.T., van der Burg B., Gustafsson J.A. (1998). Interaction of estrogenic chemicals and phytoestrogens with estrogen receptor beta. Endocrinology.

[10] Dickerson S.M., Gore A.C. (2007). Estrogenic environmental endocrine-disrupting chemical effects on reproductive neuroendocrine function and dysfunction across the life cycle. Rev. Endocr. Metab. Disord..

[11] DeRosa C., Richter P., Pohl H., Jones D.E. (1998). Environmental exposures that affect the endocrine system: public health implications. J. Toxicol. Environ. Health B Crit. Rev..

[12] Watanabe H., Suzuki A., Kobayashi M., Takahashi E., Itamoto M., Lubahn D.B., Handa H., Iguchi T. (2003). Analysis of temporal changes in the expression of estrogen-regulated genes in the uterus. J. Mol. Endocrinol..

[13] Revankar C.M., Cimino D.F., Sklar L.A., Arterburn J.B., Prossnitz E.R. (2005). A transmembrane intracellular estrogen receptor mediates rapid cell signaling. Science.

[14] Matthews J.B., Fertuck K.C., Celius T., Huang Y.W., Fong C.J., Zacharewski T.R. (2002). Ability of structurally diverse natural products and synthetic chemicals to induce gene expression mediated by estrogen receptors from various species. J. Steroid Biochem. Mol. Biol..

[15] Liu H., Zhou Q., Wang Y., Zhang Q., Cai Z., Jiang G. (2008). E-waste recycling induced polybrominated diphenyl ethers, polychlorinated biphenyls, polychlorinated dibenzo-*p*-dioxins and dibenzo-furans pollution in the ambient environment. Environ. Int..

[16] Wen S., Yang F., Li J.G., Gong Y., Zhang X.L., Hui Y., Wu Y.N., Zhao Y.F., Xu Y. (2009). Polychlorinated dibenzo-*p*-dioxin and dibenzofurans (PCDD/FS), polybrominated diphenyl ethers (PBDES), and polychlorinated biphenyls (PCBS) monitored by tree bark in an e-waste recycling area. Chemosphere.

[17] Izzotti A., Kanitz S., D’Agostini F., Camoirano A., de Flora S. (2009). Formation of adducts by bisphenol A, an endocrine disruptor, in DNA *in vitro* and in liver and mammary tissue of mice. Mutat. Res..

[18] Stavrakakis C., Hequet V., Faur C., Andres Y., Le Cloirec P., Colin R. (2008). Biodegradation of endocrine disrupters: case of 17beta-estradiol and bisphenol a. Environ. Technol..

[19] Habert R., Muczynski V., Lehraiki A., Lambrot R., Lecureuil C., Levacher C., Coffigny H., Pairault C., Moison D., Frydman R. (2009). Adverse effects of endocrine disruptors on the foetal testis development: focus on the phthalates. Folia Histochem. Cytobiol..

[20] Heudorf U., Mersch-Sundermann V., Angerer J. (2007). Phthalates: toxicology and exposure. Int. J. Hyg. Environ. Health.

[21] Han E.H., Jeong T.C., Jeong H.G. (2007). Methoxychlor suppresses the 2,3,7,8-tetrachlorodibenzo-*p*-dioxin (TCDD)-inducible CYP1A1 expression in murine Hepa-1c1c7 cells. J. Toxicol. Environ. Health A.

[22] Keum Y.S., Lee Y.H., Kim J.H. (2009). Metabolism of methoxychlor by Cunninghamella elegans ATCC36112. J. Agric. Food Chem..

[23] Schneider S., Kaufmann W., Buesen R., van Ravenzwaay B. (2008). Vinclozolin—the lack of a transgenerational effect after oral maternal exposure during organogenesis. Reprod. Toxicol..

[24] Vilela M.L., Willingham E., Buckley J., Liu B.C., Agras K., Shiroyanagi Y., Baskin L.S. (2007). Endocrine disruptors and hypospadias: Role of genistein and the fungicide vinclozolin. Urology.

[25] Hagiwara H., Sugizaki T., Tsukamoto Y., Senoh E., Goto T., Ishihara Y. (2008). Effects of alkylphenols on bone metabolism *in vivo* and *in vitro*. Toxicol. Lett..

[26] Hirose A., Koizumi M., Hasegawa R. (2000). Bisphenol. Alkylphenols. Nihon Rinsho.

[27] Hancock K.D., Coleman E.S., Tao Y.X., Morrison E.E., Braden T.D., Kemppainen B.W., Akingbemi B.T. (2009). Genistein decreases androgen biosynthesis in rat Leydig cells by interference with luteinizing hormone-dependent signaling. Toxicol. Lett..

[28] Whitten P.L., Patisaul H.B., Young L.J. (2002). Neurobehavioral actions of coumestrol and related isoflavonoids in rodents. Neurotoxicol. Teratol..

[29] Cheek A.O., Brouwer T.H., Carroll S., Manning S., McLachlan J.A., Brouwer M. (2001). Experimental evaluation of vitellogenin as a predictive biomarker for reproductive disruption. Environ. Health Perspect..

[30] Tian H., Ru S., Wang Z., Cai W., Wang W. (2009). Estrogenic effects of monocrotophos evaluated by vitellogenin mRNA and protein induction in male goldfish (*Carassius auratus*).. Comp. Biochem. Physiol. C Toxical. Pharmacol..

[31] Ebrahimi M. (2007). Vitellogenin assay by enzyme-linked immunosorbant assay as a biomarker of endocrine disruptor chemicals pollution. Pak. J. Biol. Sci..

[32] Henry T.B., McPherson J.T., Rogers E.D., Heah T.P., Hawkins S.A., Layton A.C., Sayler G.S. (2009). Changes in the relative expression pattern of multiple vitellogenin genes in adult male and larval zebrafish exposed to exogenous estrogens. Comp. Biochem. Physiol. A Mol. Integr. Physiol..

[33] Jung J.H., Shim W.J., Addison R.F., Baek J.M., Han C.H. (2006). Protein and gene expression of VTG in response to 4-nonylphenol in rockfish (Sebastes schlegeli).. Comp. Biochem. Physiol. C Toxical. Pharmacol..

[34] Rey Vazquez G., Meijide F.J., Da Cuna R.H., Lo Nostro F.L., Piazza Y.G., Babay P.A., Trudeau V.L., Maggese M.C., Guerrero G.A. (2009). Exposure to waterborne 4-tert-octylphenol induces vitellogenin synthesis and disrupts testis morphology in the South American freshwater fish *Cichlasoma dimerus* (Teleostei, Perciformes).. Comp. Biochem. Physiol. C Toxical. Pharmacol..

[35] Andersson C., Katsiadaki I., Lundstedt-Enkel K., Orberg J. (2007). Effects of 17alpha-ethynylestradiol on EROD activity, spiggin and vitellogenin in three-spined stickleback (*Gasterosteus aculeatus*).. Aquat. Toxicol..

[36] Puinean A.M., Rotchell J.M. (2006). Vitellogenin gene expression as a biomarker of endocrine disruption in the invertebrate, *Mytilus edulis*. Mar. Environ. Res..

[37] Biales A.D., Bencic D.C., Lazorchak J.L., Lattier D.L. (2007). A quantitative real-time polymerase chain reaction method for the analysis of vitellogenin transcripts in model and nonmodel fish species. Environ. Toxicol. Chem..

[38] Owens W., Koeter H.B. (2003). The OECD program to validate the rat uterotrophic bioassay: An overview. Environ. Health Perspect..

[39] Heikaus S., Winterhager E., Traub O., Grummer R. (2002). Responsiveness of endometrial genes Connexin26, Connexin43, C3 and clusterin to primary estrogen, selective estrogen receptor modulators, phyto- and xenoestrogens. J. Mol. Endocrinol..

[40] Diel P., Schulz T., Smolnikar K., Strunck E., Vollmer G., Michna H. (2000). Ability of xeno- and phytoestrogens to modulate expression of estrogen-sensitive genes in rat uterus: estrogenicity profiles and uterotropic activity. J. Steroid Biochem. Mol. Biol..

[41] Hopert A.C., Beyer A., Frank K., Strunck E., Wunsche W., Vollmer G. (1998). Characterization of estrogenicity of phytoestrogens in an endometrial-derived experimental model. Environ. Health Perspect..

[42] Sahu A., Lambris J.D. (2001). Structure and biology of complement protein C3, a connecting link between innate and acquired immunity. Immunol. Rev..

[43] Li S.H., Huang H.L., Chen Y.H. (2002). Ovarian steroid-regulated synthesis and secretion of complement c3 and factor b in mouse endometrium during the natural estrous cycle and pregnancy period. Biol. Reprod..

[44] Babal P., Ruchko M., Campbell C.C., Gilmour S.P., Mitchell J.L., Olson J.W., Gillespie M.N. (2001). Regulation of ornithine decarboxylase activity and polyamine transport by agmatine in rat pulmonary artery endothelial cells. J. Pharmacol. Exp. Ther..

[45] Dwivedi A., Gupta G., Keshri G., Dhar J.D. (1999). Changes in uterine ornithine decarboxylase activity and steroid receptor levels during decidualization in the rat induced by CDRI-85/287. Eur. J. Endocrinol..

[46] Teng C.S., Teng C.T. (1978). Studies on sex-organ development. Oestrogenic effect on ornithine decarboxylase activity in the differentiating Mullerian ducts and other organs of the chick embryo. Biochem. J..

[47] Kogo H., Johnson D.C., Dey S.K., Takeo S. (1993). A comparison of the effects of estradiol and 2- and 4-hydroxyestradiol on uterine ornithine decarboxylase activity in immature rats. Jpn. J. Pharmacol..

[48] Webster R.A., Zaloudek C.J., Inman B.C., Stewart P.J. (1984). Estrogen-like stimulation of uterine ornithine decarboxylase by cholera toxin. Am. J. Physiol..

[49] Jakowlew S.B., Breathnach R., Jeltsch J.M., Masiakowski P., Chambon P. (1984). Sequence of the pS2 mRNA induced by estrogen in the human breast cancer cell line MCF-7. Nucleic Acids Res..

[50] Zajchowski D.A., Sager R. (1991). Induction of estrogen-regulated genes differs in immortal and tumorigenic human mammary epithelial cells expressing a recombinant estrogen receptor. Mol. Endocrinol..

[51] Vivacqua A., Recchia A.G., Fasanella G., Gabriele S., Carpino A., Rago V., Di Gioia M.L., Leggio A., Bonofiglio D., Liguori A. (2003). The food contaminants bisphenol a and 4-nonylphenol act as agonists for estrogen receptor alpha in MCF7 breast cancer cells. Endocrine.

[52] Hewetson A., Chilton B.S. (1997). Molecular cloning and hormone-dependent expression of rabbit MUC1 in the cervix and uterus. Biol. Reprod..

[53] Spencer T.E., Bartol F.F., Bazer F.W., Johnson G.A., Joyce M.M. (1999). Identification and characterization of glycosylation-dependent cell adhesion molecule 1-like protein expression in the ovine uterus. Biol. Reprod..

[54] Ren L., Marquardt M.A., Lech J.J. (1997). Estrogenic effects of nonylphenol on *pS2*, *ER* and *MUC1* gene expression in human breast cancer cells-MCF-7. Chem. Biol. Interact..

[55] Gao P., Zhou G.Y., Guo L.L., Zhang Q.H., Zhen J.H., Fang A.J., Lin X.Y. (2007). Reversal of drug resistance in breast carcinoma cells by anti-mdr1 ribozyme regulated by the tumor-specific MUC-1 promoter. Cancer Lett..

[56] Bagchi I.C., Cheon Y.P., Li Q., Bagchi M.K. (2003). Progesterone receptor-regulated gene networks in implantation. Front. Biosci..

[57] Beyer C., Damm N., Brito V., Kuppers E. (2002). Developmental expression of progesterone receptor isoforms in the mouse midbrain. Neuroreport.

[58] Sauter C.N., McDermid R.L., Weinberg A.L., Greco T.L., Xu X., Murdoch F.E., Fritsch M.K. (2005). Differentiation of murine embryonic stem cells induces progesterone receptor gene expression. Exp. Cell Res..

[59] Lee Y.J., Gorski J. (1996). Estrogen-induced transcription of the progesterone receptor gene does not parallel estrogen receptor occupancy. Proc. Natl. Acad. Sci. USA.

[60] Harper N., Wang X., Liu H., Safe S. (1994). Inhibition of estrogen-induced progesterone receptor in MCF-7 human breast cancer cells by aryl hydrocarbon (Ah) receptor agonists. Mol. Cell. Endocrinol..

[61] Vo T.T., Jung E.M., Choi K.C., Yu F.H., Jeung E.B. (2011). Estrogen receptor alpha is involved in the induction of Calbindin-D_9k_ and progesterone receptor by parabens in GH3 cells: A biomarker gene for screening xenoestrogens. Steroids.

[62] Kim Y.R., Jung E.M., Choi K.C., Jeung E.B. (2011). Synergistic effects of octylphenol and isobutyl paraben on the expression of calbindin-D9k in GH3 rat pituitary cells. Int. J. Mol. Med..

[63] Schafer B.W., Heizmann C.W. (1996). The s100 family of ef-hand calcium-binding proteins: Functions and pathology. Trends Biochem. Sci..

[64] Santamaria-Kisiel L., Rintala-Dempsey A.C., Shaw G.S. (2006). Calcium-dependent and -independent interactions of the s100 protein family. Biochem. J..

[65] Jeung E.B., Krisinger J., Dann J.L., Leung P.C. (1992). Molecular cloning of the full-length cDNA encoding the human calbindin-D_9k_. FEBS Lett..

[66] Jeung E.B., Leung P.C., Krisinger J. (1994). The human calbindin-D_9k _gene. Complete structure and implications on steroid hormone regulation. J. Mol. Biol..

[67] Johansson C., Brodin P., Grundstrom T., Forsen S., Drakenberg T. (1991). Mutation of the pseudo-EF-hand of calbindin-D_9k_ into a normal EF-hand. Biophysical studies. Eur. J. Biochem..

[68] Choi K.C., Jeung E.B. (2003). The biomarker and endocrine disruptors in mammals. J. Reprod. Dev..

[69] Christakos S., Gabrielides C., Rhoten W.B. (1989). Vitamin D-dependent calcium binding proteins: Chemistry, distribution, functional considerations, and molecular biology. Endocr. Rev..

[70] Choi K.C., Jeung E.B. (2008). Molecular mechanism of regulation of the calcium-binding protein calbindin-d9k, and its physiological role(s) in mammals: A review of current research. J. Cell. Mol. Med..

[71] Darwish H.M., DeLuca H.F. (1992). Identification of a 1,25-dihydroxyvitamin D3-response element in the 5'-flanking region of the rat calbindin-D_9k_ gene. Proc. Natl. Acad. Sci. USA.

[72] Mensenkamp A.R., Hoenderop J.G., Bindels R.J. (2006). Recent advances in renal tubular calcium reabsorption. Curr. Opin. Nephrol. Hypertens..

[73] Shamley D.R., Opperman L.A., Buffenstein R., Ross F.P. (1992). Ontogeny of calbindin-D_28k_ and calbindin-D_9k_ in the mouse kidney, duodenum, cerebellum and placenta. Development.

[74] Colnot S., Romagnolo B., Lambert M., Cluzeaud F., Porteu A., Vandewalle A., Thomasset M., Kahn A., Perret C. (1998). Intestinal expression of the calbindin-d9k gene in transgenic mice. Requirement for a CDx2-binding site in a distal activator region. J. Biol. Chem..

[75] Barley N.F., Prathalingam S.R., Zhi P., Legon S., Howard A., Walters J.R. (1999). Factors involved in the duodenal expression of the human calbindin-D_9k_ gene. Biochem. J..

[76] Peng J.B., Chen X.Z., Berger U.V., Vassilev P.M., Brown E.M., Hediger M.A. (2000). A rat kidney-specific calcium transporter in the distal nephron. J. Biol. Chem..

[77] Lee G.S., Jung E.M., Choi K.C., Oh G.T., Jeung E.B. (2009). Compensatory induction of the TRPV6 channel in a calbindin-D_9k_ knockout mouse: Its regulation by 1,25-hydroxyvitamin D3. J. Cell. Biochem..

[78] Krisinger J., Dann J.L., Applegarth O., Currie W.D., Jeung E.B., Staun M., Leung P.C. (1993). Calbindin-D_9k_ gene expression during the perinatal period in the rat: Correlation to estrogen receptor expression in uterus. Mol. Cell. Endocrinol..

[79] Krisinger J., Dann J.L., Currie W.D., Jeung E.B., Leung P.C. (1992). Calbindin-D_9k_ mRNA is tightly regulated during the estrous cycle in the rat uterus. Mol. Cell. Endocrinol..

[80] L'Horset F., Blin C., Colnot S., Lambert M., Thomasset M., Perret C. (1994). Calbindin-D_9k_ gene expression in the uterus: Study of the two messenger ribonucleic acid species and analysis of an imperfect estrogen-responsive element. Endocrinology.

[81] Darwish H., Krisinger J., Furlow J.D., Smith C., Murdoch F.E., DeLuca H.F. (1991). An estrogen-responsive element mediates the transcriptional regulation of calbindin-D_9k_ gene in rat uterus. J. Biol. Chem..

[82] Lee K.Y., Oh G.T., Kang J.H., Shin S.M., Heo B.E., Yun Y.W., Paik S.G., Krisinger J., Leung P.C., Jeung E.B. (2003). Transcriptional regulation of the mouse calbindin-D_9k_ gene by the ovarian sex hormone. Mol. Cells.

[83] Hong E.J., Choi K.C., Jeung E.B. (2004). Induction of calbindin-D_9k_ messenger RNA and protein by maternal exposure to alkylphenols during late pregnancy in maternal and neonatal uteri of rats. Biol. Reprod..

[84] Dang V.H., Choi K.C., Hyun S.H., Jeung E.B. (2007). Induction of uterine calbindin-D_9k_ through an estrogen receptor-dependent pathway following single injection with xenobiotic agents in immature rats. J. Toxicol. Environ. Health A.

[85] Tatsumi K., Higuchi T., Fujiwara H., Nakayama T., Itoh K., Mori T., Fujii S., Fujita J. (1999). Expression of calcium binding protein D_9k_ messenger RNA in the mouse uterine endometrium during implantation. Mol. Hum. Reprod..

[86] Nie G.Y., Li Y., Wang J., Minoura H., Findlay J.K., Salamonsen L.A. (2000). Complex regulation of calcium-binding protein D_9k_ (calbindin-D_9k_) in the mouse uterus during early pregnancy and at the site of embryo implantation. Biol. Reprod..

[87] An B.S., Choi K.C., Kang S.K., Lee G.S., Hong E.J., Hwang W.S., Jeung E.B. (2003). Mouse calbindin-D_9k_ gene expression in the uterus during late pregnancy and lactation. Mol. Cell. Endocrinol..

[88] An B.S., Choi K.C., Kang S.K., Hwang W.S., Jeung E.B. (2003). Novel calbindin-D_9k_ protein as a useful biomarker for environmental estrogenic compounds in the uterus of immature rats. Reprod. Toxicol..

[89] An B.S., Kang S.K., Shin J.H., Jeung E.B. (2002). Stimulation of calbindin-D_9k_ mRNA expression in the rat uterus by octyl-phenol, nonylphenol and bisphenol. Mol. Cell. Endocrinol..

[90] Dang V.H., Choi K.C., Hyun S.H., Jeung E.B. (2007). Analysis of gene expression profiles in the offspring of rats following maternal exposure to xenoestrogens. Reprod. Toxicol..

[91] Dang V.H., Choi K.C., Jeung E.B. (2007). Tetrabromodiphenyl ether (BDE 47) evokes estrogenicity and calbindin-D_9k_ expression through an estrogen receptor-mediated pathway in the uterus of immature rats. Toxicol. Sci..

[92] Hong E.J., Ji Y.K., Choi K.C., Manabe N., Jeung E.B. (2005). Conflict of estrogenic activity by various phthalates between *in vitro* and *in vivo* models related to the expression of calbindin-D_9k_. J. Reprod. Dev..

[93] Lee G.S., Choi K.C., Kim H.J., Jeung E.B. (2004). Effect of genistein as a selective estrogen receptor beta agonist on the expression of calbindin-d9k in the uterus of immature rats. Toxicol. Sci..

[94] Vo T.T., Jeung E.B. (2009). An evaluation of estrogenic activity of parabens using uterine calbindin-D_9k_ gene in an immature rat model. Toxicol. Sci..

[95] Lee G.S., Kim H.J., Jung Y.W., Choi K.C., Jeung E.B. (2005). Estrogen receptor alpha pathway is involved in the regulation of calbindin-D_9k_ in the uterus of immature rats. Toxicol. Sci..

[96] Jung E.M., An B.S., Choi K.C., Jeung E.B. (2012). Potential estrogenic activity of triclosan in the uterus of immature rats and rat pituitary GH3 cells. Toxicol. Lett..

[97] Shin J.H., Moon H.J., Kang I.H., Kim T.S., Lee S.J., Oh J.Y., Lee Y.J., Hong E.J., Jeung E.B., Han S.Y. (2007). Calbindin-D_9k_ mRNA expression in the rat uterus following exposure to methoxychlor: A comparison of oral and subcutaneous exposure. J. Reprod. Dev..

[98] Fujimoto N., Igarashi K., Kanno J., Inoue T. (2004). Identification of estrogen-responsive genes in the GH3 cell line by cDNA microarray analysis. J. Steroid Biochem. Mol. Biol..

[99] Dang V.H., Choi K.C., Jeung E.B. (2009). Estrogen receptors are involved in xenoestrogen induction of growth hormone in the rat pituitary gland. J. Reprod. Dev..

[100] Dang V.H., Nguyen T.H., Choi K.C., Jeung E.B. (2007). A calcium-binding protein, calbindin-D_9k_, is regulated through an estrogen-receptor mediated mechanism following xenoestrogen exposure in the GH3 cell line. Toxicol. Sci..

[101] Yang H., Nguyen T.T., An B.S., Choi K.C., Jeung E.B. (2011). Synergistic effects of parabens on the induction of calbindin-D_9k_ gene expression act via a progesterone receptor-mediated pathway in GH3 cells. Hum. Exp. Toxicol..

